# Outcome Predictors in Progressive Multifocal Leukoencephalopathy Associated With Multiple Sclerosis Treatments: A Multicenter Cohort Study

**DOI:** 10.1212/NXI.0000000000200558

**Published:** 2026-02-20

**Authors:** Julie Céline Blant, Nicola De Rossi, Ralf Gold, Aude Maurousset, Markus Kraemer, Lucía Romero-Pinel, Tatsuro Misu, Jean-Christophe Ouallet, Maud Pallix Guyot, Simonetta Gerevini, Christos Bakirtzis, Raquel Piñar Morales, Benjamin Vlad, Panajotis Karypidis, Xavier Moisset, Tobias J. Derfuss, Ilijas Jelcic, Guillaume Martin-Blondel, Ilya Ayzenberg, Corey McGraw, David Axel Laplaud, Christine Lebrun-Frenay, Renaud A. Du Pasquier, Raphael Bernard-Valnet, for the CORPUS and Italian PML study groups

**Affiliations:** 1 Service of Neurology, Department of Clinical Neurosciences, Lausanne University Hospital (Centre Hospitalier Universitaire Vaudois) and University of Lausanne, Switzerland;; 2 Regional Multiple Sclerosis Center, ASST-Spedali Civili dii Brescia, Montichiari, Italy;; 3 Department of Neurology St. Josef-Hospital, Ruhr University Bochum, Germany;; 4 Centre hospitalier régional universitaire de Tours, Hôpital Bretonneau, Service de neurologie, France;; 5 Department of Neurology, Alfried Krupp von Bohlen und Halbach Hospital, Essen, Germany;; 6 Department of Neurology, Medical Faculty, Heinrich Heine University of Düsseldorf, Germany;; 7 Neurology Department, Multiple Sclerosis Unit, Hospital Universitari de Bellvitge, IDIBELL, Barcelona, Spain;; 8 Department of Neurology, Tohoku University Hospital, Japan;; 9 Service de Neurologie, Pôle des Neurosciences Cliniques, CHU de Bordeaux Pellegrin Tripode, France;; 10 Service de Neurologie et Unité Neurovasculaire, Centre Hospitalier Régional d'Orléans, France;; 11 Unit of Neuroradiology, Papa Giovanni XXIII Hospital, Bergamo, Italy;; 12 Multiple Sclerosis Center, Second Department of Neurology, Aristotle University of Thessaloniki, Greece;; 13 Servicio de Neurología, Hospital Universitario Clínico San Cecilio, Granada, Spain;; 14 Department of Neurology, University Hospital Zürich and University of Zürich, Switzerland;; 15 Neurologic Clinic and Policlinic and Research Center for Clinical Neuroimmunology and Neuroscience, Departments of Medicine, Biomedicine, and Clinical Research, University Hospital Basel, University of Basel, Switzerland;; 16 Service de Neurologie, Université Clermont Auvergne, CHU de Clermont-Ferrand, Inserm, Neuro-Dol, Clermont-Ferrand, France;; 17 University Hospital of Toulouse, Infectious and Tropical Diseases Unit, France;; 18 Department of Neurology, State University of New York Upstate Medical University, Syracuse;; 19 CHU Nantes, Service de Neurologie, CRC-SEP, Nantes Université, INSERM, CIC 1413, Center for Research in Transplantation and Translational Immunology, UMR, France;; 20 Neurology, UR2CA_URRIS, Centre Hospitalier Universitaire Pasteur2, Université Nice Côte d'Azur, France; and; 21 Department of Neurology with Institute of Translational Neurology, University Hospital Münster, Germany.

## Abstract

**Background and Objectives:**

JC virus (JCV) reactivation causing progressive multifocal leukoencephalopathy (PML) is a complication in patients with multiple sclerosis (MS) treated with disease-modifying therapies (DMTs). Although natalizumab (NTZ) is most frequently involved, PML also occurs less commonly with sphingosine-1-phosphate receptor modulators (S1P-RM), dimethyl fumarate (DMF), and ocrelizumab. This study aimed to identify factors predicting worse outcomes, focusing on the influence of PML-immune reconstitution inflammatory syndrome (PML-IRIS), plasma exchange (PlEx), corticosteroids, and DMT reintroduction.

**Methods:**

This retrospective multicenter cohort study analyzed patients with MS who had JCV-associated pathology (PML or granule cell neuronopathy) from 42 centers (2009–2022). The primary outcome was disability at 12 months, measured by the modified Rankin Scale (mRS). Multivariable analyses identified predictors of poor outcomes, PML-IRIS development, and recurrent MS activity.

**Results:**

Of 96 identified patients, 94 were analyzed. Most cases occurred under NTZ (77%), followed by S1P-RM (22%) and DMF (1%). Twelve-month survival was 91.5%, with a median mRS of 3 [IQR: 2–4]. Multivariable analysis showed that higher pre-PML disability (OR: 1.95 [95% CI 1.46–2.60], *p* < 0.001), elevated CSF JCV viral load (OR: 2.45 [95% CI 1.55–3.87], *p* < 0.001), and symptomatic presentation at onset (OR: 3.93 [95% CI 1.23–12.55], *p* = 0.021) were associated with worse outcomes. Conversely, PML-IRIS was associated with better outcomes (OR: 0.28 [95% CI 0.09–0.86], *p* = 0.025). PlEx and corticosteroid use had no negative effect.

**Discussion:**

This study provides valuable insights into the management of iatrogenic PML in patients with MS. The findings may guide clinicians in making informed decisions, particularly regarding the use of PlEx, corticosteroids, and the management of PML-IRIS.

## Introduction

Progressive multifocal leukoencephalopathy (PML) is a neurologic infectious complication arising from JC virus (JCV) reactivation in the context of immunosuppression. Historically, PML was primarily associated with hematologic malignancies and HIV infection. However, the landscape has shifted, with a growing proportion of PML cases now linked to immunosuppressive treatments used in inflammatory diseases.^1^ As the primary cause of iatrogenic PML in multiple sclerosis (MS), natalizumab ([NTZ]–Tysabri, Biogen, Cambridge, MA) has been extensively studied over the past years. More recently, other disease-modifying therapies (DMTs) such as sphingosine-1-phosphate receptor modulators (S1P-RM–Gilenya and Mayzent, Novartis, Basel, Switzerland; Zeposia, Bristol Myers Squibb, Princeton, NJ) and dimethyl-fumarate (Tecfidera, Biogen, Cambridge, MA) have been implicated in PML cases,^2^ although their incidence and the breadth of related scientific research remain considerably lower in comparison with NTZ.

Despite the general observation that NTZ-associated PML tends to have a more favorable prognosis compared with other causes of immunosuppression, its mortality rate persists at approximately 10%–20%.^3,4^ Several patient characteristics have been identified as prognostic factors of survival for NTZ-associated PML, including preexisting disability levels, age at onset, symptomatic presentation, widespread lesions, and high JCV load at onset.^5-9^ Yet, research focusing on patient survival often overshadows the considerable burden of disability that survivors face, especially in the long term.^10^

The management of PML currently focuses on reestablishing a JCV-specific immune response within the brain, necessitating the discontinuation of immunosuppressive treatments. Plasma exchanges (PlEx) can aid in accelerating NTZ clearance and are commonly used in clinical practice, but their efficacy in improving survival or functional outcomes has been debated and probably depends on how early PlEx is applied after last NTZ infusion.^11-13^ Similarly, corticosteroids have shown no benefit in preventing immune reconstitution inflammatory syndrome (IRIS) and may even hinder immune responses against JCV.^13,14^ However, their use in patients exhibiting signs of IRIS has been suggested to reduce morbidity and mortality.^12,15^

Furthermore, immune restoration efforts may be complicated by PML-immune reconstitution inflammatory syndrome (PML-IRIS), which presents as clinical deterioration linked to inflammatory signs indicative of an exaggerated immune response. Although IRIS is frequently observed in NTZ-associated PML, it appears less common in other DMT-associated cases, particularly S1P-RM.^16^ Its precise repercussion on prognosis remains unclear but may contribute to additional morbidity and mortality.^6,10,15^

Finally, MS recurrence has been described after NTZ and S1P-RM withdrawal.^17,18^ To date, no instances of PML exacerbation have been documented on DMT reintroduction, even in cases where JC viral load in CSF remains low but detectable.^10,19^

Considering the rapid and promising expansion of treatments used in MS and the dissatisfying prognosis of opportunistic diseases such as PML, our objective is to define the factors that influence the outcome of DMT-associated PML. We then aim to especially assess the exact contribution of PML-IRIS, corticosteroids, and PlEx to the 12 months disability. In parallel, we would assess if some factor may predict the occurrence of IRIS and MS-recurring activity.

## Methods

### Design, Setting, and Participants

We conducted a retrospective cohort study collecting data from consecutive JCV infections (either PML or granule cell neuronopathy) in the context of treatment with MS DMT. This cohort was assembled for a previous study aiming at comparing S1P-RM and NTZ associated PML.

We included all MS adults patients who developed confirmed, probable, or possible PML based on the 2013 American Academy of Neurology criteria^20^ or JCV granule cell neuronopathy (GCN) and admitted at participating centers between 2009 and 2022. Patients with a high proportion (>40%) of missing variables were excluded.

### Outcome Measures

The main objective was to identify factors associated with the functional outcome at 12 months, assessed using the modified Rankin Scale (mRS). Secondary objectives included identifying predictors of PML-IRIS, defined as paradoxical clinical deterioration of PML following a period of stability, associated with signs of immune reconstitution, such as gadolinium-enhancing lesions, edema or mass effect, or inflammatory infiltrates on biopsy. An additional aim was to identify predictors of MS recurrence within the first year following PML, defined as the emergence of new neurologic symptoms lasting more than 24 hours without evidence of PML worsening and/or the appearance or progression of typical MS lesions on MRI.

For certain analyses, patients were classified into 2 groups based on their functional outcomes at 12 months: mRS ≤3 would be considered as a favorable outcome as patients still have preserved ambulation and self-care abilities. Conversely, mRS >3 would be considered as an unfavorable outcome.

### Statistical Analysis

Patient characteristics were described using median (interquartile range, IQR 25%–75%) for continuous variables and frequencies (n, %) for categorical variables. Comparisons between the outcome groups were conducted using Pearson χ^2^ or Fisher exact test for categorical variables and the Wilcoxon rank-sum test for continuous variables. All *p* values were 2-sided, with statistical significance set at *p* < 0.05.

For outcome analyses, we used ordinal logistic regression for mRS at 12 months and binomial logistic regression for PML-IRIS occurrence and MS recurrence. Variables identified as significant in univariate analyses and those deemed clinically important were included in the multivariable models. Missing values were imputed using a K-Nearest Neighbors approach. To assess potential collinearity, variance inflation factors were calculated and found to be acceptable (<10) for all predictors, although the expected collinearity between Expanded Disability Status Scale (EDSS) and mRS was noted (eFigure 1). Then, EDSS and mRS were not concomitantly included in the multivariable analyses.

All analyses were conducted using R (R Foundation for Statistical Computing, Vienna, Austria), using packages such as *ggplot2*, *VIM*, *forestplotter*, *gtsummary*, *flextable, party*, and *dplyr*.

### Standard Protocol Approvals, Registrations, and Patient Consents

The study was approved by the local ethics committee (Commission cantonale d'éthique de la recherche sur l'être humain) under authorization number 2021-01163. A consent waiver was obtained for deceased patients and those lost at follow-up.

### Data Availability

Owing to restrictions imposed by the ethics committee, the data supporting the findings of this study cannot be made publicly available. However, anonymized data may be shared on reasonable request with qualified, noncommercial entities, contingent on the signing of a data transfer agreement. The analysis code is openly available at: github.com/rbernardvalnet/PML_predictors.git.

## Results

**Figure 1:**
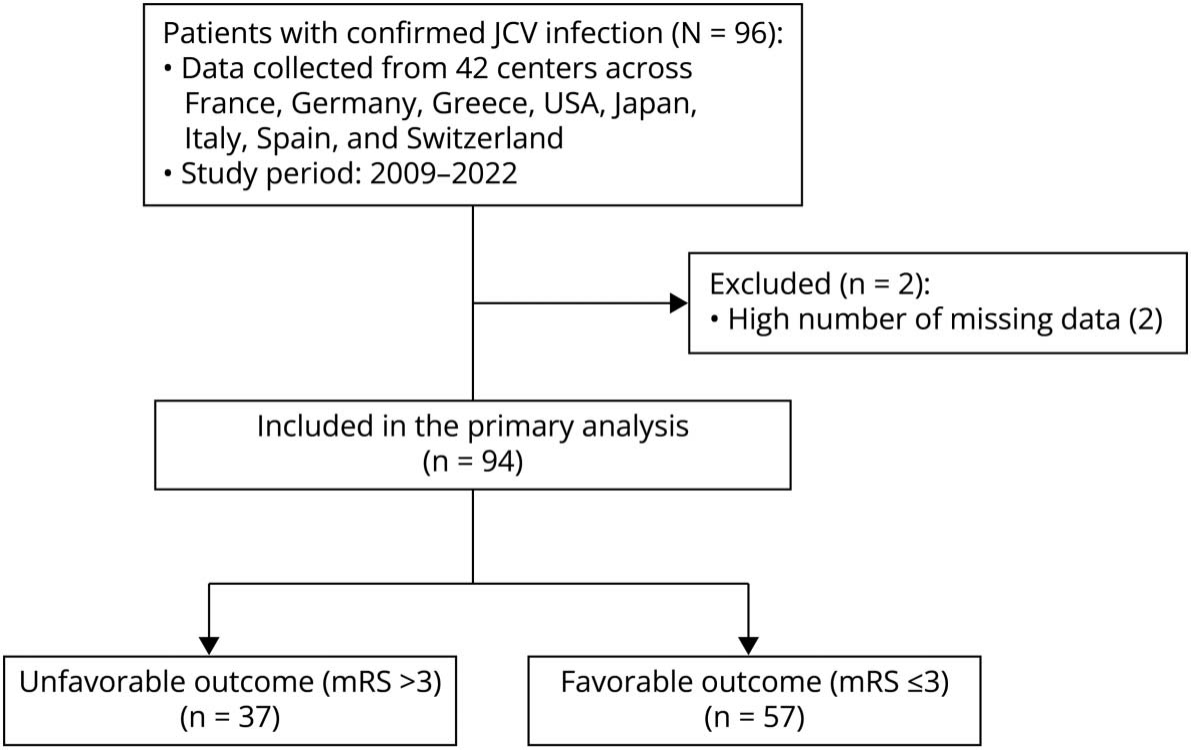
Flowchart JCV = JC virus; mRS = modified Rankin Scale.

We collected data from 96 participants diagnosed with JCV-related disease following DMTs for MS between 2009 and 2022. Two participants were excluded due to extensive missing data (Figure 1).

The final cohort comprised 94 patients from 42 international centers across 8 countries: Italy (22 centers, 35 patients), Switzerland (3 centers, 28 patients), France (7 centers, 21 patients), Germany (2 centers, 3 patients), Spain (2 centers, 2 patients), Greece (1 center, 2 patients), the United States (1 center, 2 patients), and Japan (1 center, 1 patient).

Most patients (n = 72, 77%) developed PML during NTZ treatment, with fewer cases linked to S1P-RM (n = 21%, 22%) and dimethyl fumarate (DMF, n = 1%, 1%). Notably, only 1 patient (1.1%) presented with JCV granule cell neuronopathy; all others had classical PML. In addition, 1 case of carry-over NTZ-associated PML was identified in a patient subsequently receiving S1P-RM therapy.

### Functional Outcome After PML

To assess 12-month outcomes, we categorized patients into groups with favorable (mRS ≤ 3; n = 57) and unfavorable outcomes (mRS >3; n = 37). Patients achieving favorable outcomes had distinct baseline characteristics compared with those with unfavorable outcomes, particularly regarding sex, MS subtype, disease duration, and preexisting disability (Table 1). Indeed, favorable outcomes were more common among male patients (33% vs 14%, *p* = 0.031), patients with relapsing-remitting MS (97% vs 75%, *p* = 0.012), and those with shorter disease duration (median [IQR_25-75_]: 154 [108–191] vs 204 [134–240] months, *p* = 0.018). Lower pre-PML disability, assessed through EDSS (median [IQR_25-75_]: 2.75 [2–4] vs 6 [2.75–6.75], *p* < 0.001) or mRS (median [IQR_25-75_]: 1.0 [1.0–2.0] vs 3.0 [1.0–4.0], *p* < 0.001), strongly correlated with better outcomes.

**Table 1:** Baseline Characteristics and PML Initial Presentation

	mRS at 12 mo				
Variable	N	Overall N = 94^a^	>3 N = 37^a^	≤3 N = 57^a^	*p* Value
Demographical characteristics					
Sex, female, n (%)	94	70 (74)	32 (86)	38 (67)	0.031^b^
Age at PML onset, median (Q1–Q3)	94	46 (39–51)	46 (41–52)	45 (39–51)	0.64^c^
MS history					
Type of MS, n (%)	61				0.012^d^
RRMS		54 (89)	18 (75)	36 (97)	
SPMS		7 (11)	6 (25)	1 (2.7)	
MS duration before PML, in mo, median (Q1–Q3)	90	164 (110–215)	204 (134–240)	154 (108–191)	0.018^c^
History of immunomodulator use, n (%)	94	87 (93)	35 (95)	52 (91)	0.70^d^
History of immunosuppressor use, n (%)	94	25 (27)	9 (24)	16 (28)	0.69^b^
Activity under DMT in the last 2 years, n (%)	51	15 (29)	8 (38)	7 (23)	0.25^b^
EDSS before PML, median (Q1–Q3)	90	3.25 (2.00–5.50)	6.00 (2.75–6.75)	2.75 (2.00–4.00)	<0.001^c^
mRS before PML, median (Q1–Q3)	94	2.00 (1.00–3.00)	3.00 (1.00–4.00)	1.00 (1.00–2.00)	<0.001^c^
PML presentation					
Treatment associated with PML, n (%)	94				0.77^d^
DMF		1 (1.1)	0 (0)	1 (1.8)	
Natalizumab		72 (77)	30 (81)	42 (74)	
S1P-RM		21 (22)	7 (19)	14 (25)	
Duration of treatment before PML, in mo, median (Q1–Q3)	91	46 (30–61)	49 (35–61)	42 (27–66)	0.19^c^
PML presentation, n (%)	94				0.26^b^
Asymptomatic		18 (19)	5 (14)	13 (23)	
Symptomatic		76 (81)	32 (86)	44 (77)	
Classification of PML, n (%)	94				0.60^b^
Definite		55 (59)	24 (65)	31 (54)	
Possible		18 (19)	6 (16)	12 (21)	
Probable		21 (22)	7 (19)	14 (25)	
Gd + lesion at onset (iPML), n (%)	94	45 (48)	16 (43)	29 (51)	0.47^b^
Affected lobes, n, median (Q1–Q3)	92	2.00 (1.00–4.00)	3.00 (2.00–4.00)	2.00 (1.00–3.00)	0.13^c^
Infratentorial involvement, n (%)	94	27 (29)	14 (38)	13 (23)	0.12^b^
Oedema at PML onset, n (%)	61	10 (16)	5 (21)	5 (14)	0.49^d^
CSF JCV load, median (Q1–Q3)	86	312 (28–3,000)	1,545 (64–6,001)	100 (15–868)	0.003^c^
Time from PML first suspicion to diagnosis, in d, median (Q1–Q3)	94	20 (8–67)	20 (10–42)	19 (8–70)	0.70^c^

Abbreviations: DMF = dimethyl fumarate; DMT = Disease-modifying-treatments; JCV = JC virus; mRS = modified Rankin Scale; MS = multiple sclerosis; PML = progressive multifocal leukoencephalopathy; RRMS = relapsing remitting multiple sclerosis; S1P-RM = sphingosine-1-phosphate receptor modulator; SPMS = secondary progressive multiple sclerosis.

a Median (IQR) or Frequency (%).

b Pearson χ^2^ test.

c Wilcoxon rank-sum test.

d Fisher exact test.

At diagnosis, patients with favorable outcomes demonstrated lower JCV loads in CSF (median [IQR_25-75_]: 100 [15–868] vs 1,545 [64–6,001] copies/mL, *p* = 0.003), despite similar clinical and radiologic presentations (Table 1).

Acute management strategies were consistent between outcome groups (Table 2) with similar rates of PML-IRIS (73% vs 82%, *p* = 0.27) and corticosteroid administration (81% vs 70%, *p* = 0.24). In NTZ-associated cases, PlEx use was comparable (78.6% vs 80%, *p* = 0.999).

**Table 2:** PML Course and Treatment

	mRS at 12 mo				
Variable	N	Overall N = 941^a^	>3 N = 371^a^	≤3 N = 571^a^	*p* Value
PML management					
Delay from PML presumption to treatment withdrawal, in d, median (Q1–Q3)	83	4 (0–17)	2 (0–23)	7 (0–15)	0.70^b^
PML treatments					
Plasma exchanges, n (%)	94	62 (66)	28 (76)	34 (60)	0.11^c^
Mefloquine, n (%)	94	28 (30)	14 (38)	14 (25)	0.17^c^
Mirtazapine, n (%)	94	43 (46)	19 (51)	24 (42)	0.38^c^
PML-IRIS					
Occurrence, n (%)	94	74 (79)	27 (73)	47 (82)	0.27^c^
IRIS/iPML treatments					
Maraviroc, n (%)	93	20 (22)	5 (14)	15 (27)	0.13^c^
Corticosteroids, n (%)	94	70 (74)	30 (81)	40 (70)	0.24^c^
MS after PML					
MS activity in the 12 first months, n (%)	94	38 (40)	12 (32)	26 (46)	0.20^c^
MS DMT introduction after PML, n (%)	84	50 (60)	14 (41)	36 (72)	0.005^c^
Clinical outcome					
mRS at 12 mo, median (Q1–Q3)	94	3.00 (2.00–4.00)	4.00 (4.00–5.00)	2.00 (2.00–3.00)	<0.001^b^
Death within 12 months, n (%)	94	8 (8.5)	8 (22)	0 (0)	<0.001^d^
Last mRS, median (Q1–Q3)	94	3.00 (2.00–4.00)	4.00 (4.00–6.00)	3.00 (2.00–3.00)	<0.001^b^
Follow-up duration, median (Q1–Q3)	94	17 (12–41)	21 (7–41)	14 (12–43)	0.45^b^

Abbreviations: DMT = Disease-modifying treatments; iPML = inflammatory PML; IRIS = immune reconstitution inflammatory syndrome; mRS = modified Rankin Scale; MS = multiple sclerosis; PML = progressive multifocal leukoencephalopathy.

a Median (IQR) or Frequency (%).

b Wilcoxon rank-sum test.

c Fisher exact test.

d Pearson χ^2^ test.

Reintroduction of DMTs post-PML was more prevalent among patients with favorable outcomes (72% vs 41%, *p* = 0.005). However, recurrence of MS activity post-PML was similar between groups (46% vs 32%, *p* = 0.20). Importantly, most patients experienced lasting PML-related disabilities, reflected by elevated mRS scores at follow-up (median [IQR_25-75_]: 3.0 [2.0–3.0] vs 4.0 [4–6], *p* < 0.001).

**Figure 2:**
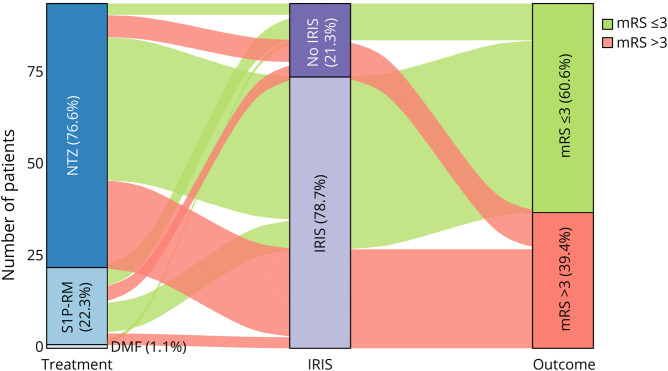
Individual Trajectories in Patients With PML Associated With MS DMT Alluvial plot showing individual patient trajectories based on the DMT associated with PML, the occurrence of IRIS, and the 12-month outcome as measured by the mRS. DMT = disease-modifying treatments; IRIS = immune reconstitution inflammatory syndrome; mRS = modified Rankin Scale; MS = multiple sclerosis; PML = progressive multifocal leukoencephalopathy.

Figure 2 illustrates individual patient trajectories, highlighting differences in outcomes by treatment type and PML-IRIS status.

**Figure 3:**
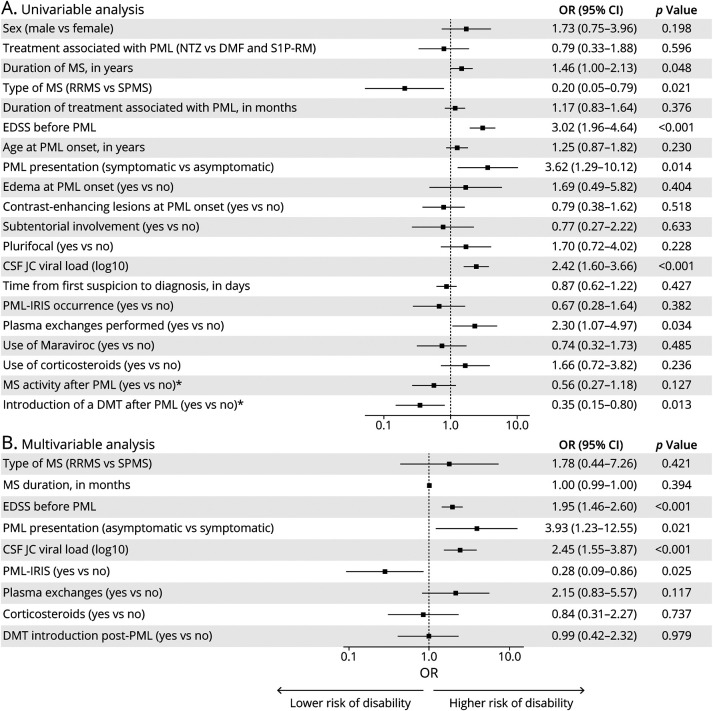
Predictors of Disability (mRS) at 12 Months After PML Onset Forest plots representing the effect of predefined predictors on disability, assessed using the mRS at 12 months following disease onset, shown for univariate analysis (A) and multivariable ordinal regression (B). *Within the first 12 months after PML diagnosis. DMF = dimethyl fumarate; DMT = disease-modifying treatments; EDSS = Expanded Disability Status Scale; IRIS = immune reconstitution inflammatory syndrome; JCV = JC virus; mRS = modified Rankin Scale; MS = multiple sclerosis; NTZ = natalizumab; PML = progressive multifocal leukoencephalopathy; RRMS = relapsing-remitting MS; S1P-RM = sphingosine-1-phosphate receptor modulators; SPMS = secondary progressive MS.

To elucidate factors contributing to unfavorable outcomes, univariable ordinal regression was conducted (Figure 3A). Factors associated with increased disability risk included prolonged MS duration (unadjusted OR: 1.46 [1.00–2.13], *p* = 0.048), higher pre-PML disability (unadjusted OR: 3.02 [1.96–4.64], *p* < 0.001), progressive MS subtype (unadjusted OR: 5.0 [1.27–20], *p* = 0.021), elevated initial JCV load in CSF (unadjusted OR: 2.42 [1.60–3.66], *p* < 0.001), symptomatic clinical onset (unadjusted OR: 3.62 [1.29–10.12], *p* = 0.014), and PlEx therapy (unadjusted OR: 2.30 [1.07–4.97], *p* = 0.034). Conversely, DMT reintroduction post-PML reduced disability risk (unadjusted OR: 0.35 [0.15–0.80], *p* = 0.013).

Multivariable ordinal regression analysis, incorporating corticosteroid treatment and the occurrence of PML-IRIS, identified several independent predictors of worse outcomes (Figure 3B). These included higher pre-PML disability (EDSS; adjusted OR: 1.95 [95% CI 1.46–2.60], *p* < 0.001), symptomatic onset (adjusted OR: 3.93 [1.23–12.55], *p* = 0.021), and elevated initial CSF JCV viral load (adjusted OR: 2.45 [1.55–3.87], *p* < 0.001). A conditional regression tree further delineated thresholds associated with poor prognosis, with EDSS >5.5 and CSF JCV viral load >3.6 log_10_ copies/mL identifying patients at higher risk (eFigure 2). Notably, the occurrence of PML-IRIS was independently associated with a favorable outcome (adjusted OR: 0.28 [0.09–0.86], *p* = 0.025).

Neither corticosteroids (adjusted OR: 0.84 [0.31–2.27], *p* = 0.737) nor PlEx (adjusted OR: 2.15 [0.83–5.57], *p* = 0.117) influenced disability outcomes, even among NTZ-treated patients alone (adjusted OR: 1.00 [0.31–3.17], *p* = 0.995).

### Predictors of PML-IRIS Development

**Figure 4:**
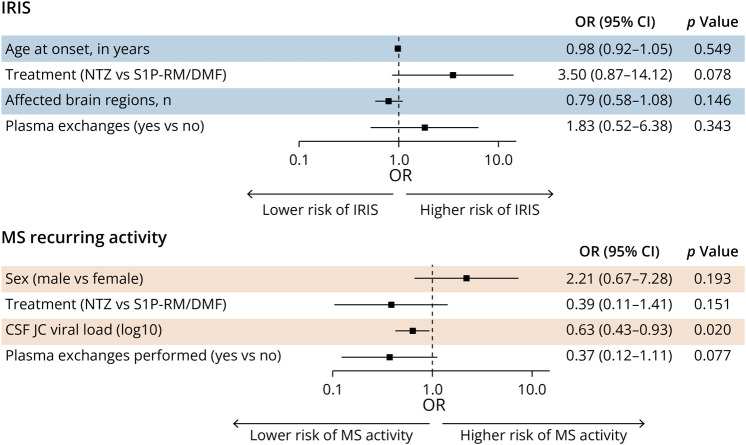
Predictors of IRIS and MS Activity Within 12 Months After PML Forest plots representing the effect of predefined predictors on (A) the risk of developing PML-IRIS and (B) the occurrence of recurring MS activity, based on multivariable binomial regression analysis. DMF = dimethyl fumarate; IRIS = immune reconstitution inflammatory syndrome; MS = multiple sclerosis; NTZ = natalizumab; S1P-RM = sphingosine-1-phosphate receptor modulators.

We aimed to identify variables present at disease onset that could predict the occurrence of PML-IRIS and to evaluate whether the initial management strategy for PML influenced this risk. Using a binomial univariable analysis, we confirmed that NTZ treatment increased the risk of developing PML-IRIS (unadjusted OR: 7.00 [2.36–20.80], *p* < 0.001), consistent with our previous findings.^21^ Conversely, older age (unadjusted OR: 0.58 [0.35–0.98], *p* = 0.043) and a higher number of affected brain regions at PML onset (unadjusted OR: 0.71 [0.53–0.96], *p* = 0.026) were associated with a reduced risk (eFigure 3). Although PlEx (unadjusted OR: 4.05 [1.44–11.4], *p* = 0.008) initially appeared to correlate with an increased risk of PML-IRIS, none of these parameters remained significant in the subsequent binomial multivariable model, suggesting the presence of confounding factors (Figure 4A).

Similarly, when analyzing the effect of PlEx on PML-IRIS exclusively within the NTZ-treated subgroup, no association was found (adjusted OR: 2.06 [0.44–9.74], *p* = 0.362). However, given the repercussion of PlEx timing on the pharmacokinetics of NTZ and the subsequent restoration of an immune response that may trigger IRIS, we further explored this aspect.^11,22^ In our NTZ-treated patients, the delay between the last NTZ infusion and PlEx was available for 35 patients (48.2%), with a median interval of 15 days (interquartile range [IQR] 25–75: 5–35). We then conducted a univariate analysis (due to limited sample size) comparing early (<4 weeks, n = 23, 65.7%) vs late (≥4 weeks, n = 12, 34.3%) PlEx. Early PlEx was not associated with an unfavorable outcome (mRS >3: 11/23, 47.8% vs 6/12, 50%; *p* = 0.921), but it was associated with a higher risk of developing PML-IRIS (23/23, 100% vs 9/12, 75%; *p* = 0.015).

### Predictors of MS Recurrence Post-PML

MS recurring activity was frequent after PML, with 40% of patients presenting radiologic or clinical activity (Table 2). Consequently, in most cases (60%), an MS DMT was reintroduced to control disease activity after a median of 6.9 months following PML onset (IQR _25–75_: 4.0–11.1), with 46% restarted within the first 12 months. The treatments initiated were predominantly platform therapies (glatiramer acetate, interferon-β, teriflunomide, or DMF), used in 98% of these patients.

We then tried to delineate predictors of recurring MS activity following PML. Univariable analysis revealed a higher risk of recurrence in female patients (unadjusted OR: 3.39 [1.14–10.09], *p* = 0.028). By contrast, NTZ treatment (unadjusted OR: 0.29 [0.11–0.78], *p* = 0.014), elevated initial CSF JCV load (unadjusted OR: 0.51 [0.31–0.84], *p* = 0.009), and PlEx therapy (unadjusted OR: 0.20 [0.08–0.50], *p* < 0.001) were associated with a lower risk of MS recurrence (eFigure 4). Multivariable analysis confirmed that a higher initial CSF JCV load remained independently protective against MS recurrence (adjusted OR: 0.63 [0.43–0.93], *p* = 0.020) (Figure 4B).

## Discussion

PML occurring in the iatrogenic context of MS DMT remains a challenge in terms of management, and several questions remain unanswered—particularly concerning the beneficial or detrimental roles of corticosteroids and PlEx, as well as the repercussion of PML-IRIS on prognosis.

In this study, we leveraged a large multicenter cohort of iatrogenic PML and other forms of JCV infection to better elucidate the parameters influencing outcome. Our findings confirmed that pre-PML disability, symptomatic presentation, and higher CSF JCV load at PML onset were predictors of a worse outcome 12 months after onset. Notably, we found that the occurrence of PML-IRIS was associated with a reduced risk of long-term disability.

We were unable to demonstrate a significant overall influence of PlEx on either PML outcome or the risk of developing PML-IRIS. However, data from a limited number of patients suggest that early administration of PlEx may be associated with an increased risk of developing PML-IRIS. Similarly, we did not identify reliable predictors of PML-IRIS or of recurrent MS activity, with the exception of the initial CSF JCV load, which was associated with the latter.

In our cohort, 12-month survival was 91.5%, slightly higher than previously reported.^3,4^ This may reflect evolving practices in the management of MS-associated PML in more recent cohorts notably due to the implementation of risk mitigation strategies, such as radiologic surveillance in at-risk patients.^7^ Furthermore, we would like to point out that previous data, particularly those derived from the Biogen safety database,^5^ were obtained in patients with confirmed JCV positivity (CSF or biopsy), which likely reflects a more severe clinical course.

While previous studies focused primarily on survival, we adopted a slightly different approach by analyzing disability as measured by the mRS. Nevertheless, we were able to replicate most previously identified prognostic factors in NTZ-treated patients, such as pre-PML disability, symptomatic onset, and CSF viral load at diagnosis.^5,8^ However, neither age nor the extent of brain lesion dissemination was found to be significant in our analysis.

These results underscore the importance of detecting PML early in the disease course, ideally at the asymptomatic stage. JC virus replication, as reflected by CSF viral load, remains a major prognostic factor, as repeatedly demonstrated in PML. Indeed, JCV DNA in CSF likely reflects the active disease process and typically declines following immune recovery, supporting its use as a surrogate marker.^23^

The contribution of PML-IRIS to PML outcomes has been widely debated. Although immune recovery is essential for JCV control and early inflammatory signs are associated with improved survival,^24,25^ PML-IRIS can provoke clinical deterioration and, in rare cases, be life-threatening.^26^ In HIV-associated PML, its repercussion remains controversial, with some studies showing improved survival in patients developing IRIS^26^ and others reporting similar outcomes between groups.^27,28^ In NTZ-associated PML, previous studies have not demonstrated a significant influence of IRIS on outcome. By contrast, we found that PML-IRIS was associated with better outcomes in a large proportion of MS-associated PML cases. This may partially explain the relatively favorable prognosis of patients with MS who develop PML compared with other populations.

Regarding the consequences of PML management on outcomes, we confirmed the absence of both beneficial and detrimental effects of PlEx in NTZ-associated PML.^12^ While previous studies have suggested that PlEx may prolong PML-IRIS,^13^ we did not observe an increased incidence of IRIS with its use. However, this finding may be biased by the fact that some patients received PlEx at a later stage. T-cell trafficking is known to correlate closely with α4-integrin saturation by NTZ,^11,22^ and a rapid desaturation induced by early PlEx could trigger abrupt immune restoration, potentially favoring the onset of IRIS—as observed in a subgroup of patients with available timing data.

Similarly, we found no effect of corticosteroids on PML outcome. However, we could not distinguish whether corticosteroids were administered prophylactically or as treatment for IRIS. This distinction is important, as corticosteroids may suppress anti-JCV T-cell responses and thereby hinder the beneficial immune response in PML.^14^

Surprisingly, when we explored predictors of recurrent MS activity, we found that higher JC viral load was associated with a lower risk of recurrence. This counterintuitive finding may partly reflect a bias in the follow-up and assessment of MS activity in patients with greater disability. Alternatively, one could hypothesize that high levels of JCV replication modulate overall CNS immune responses. Indeed, as observed in many viral infections, JCV may induce T-cell exhaustion. PML patients have been shown to exhibit an upregulation of inhibitory molecules such as PD-1 on both CD4^+^ and CD8^+^ T cells (not limited to JCV-specific populations).^29,30^ We therefore speculate that in patients with the highest levels of viral replication, this enhanced inhibitory signaling may impair T-cell effector function and consequently decrease MS inflammatory activity.^31^ Despite the strengths of a sizable, multicenter cohort, the retrospective nature of this study and reliance on heterogeneous clinical records introduce potential selection and information bias, especially given variable diagnostic and treatment protocols across 42 centers in 8 countries. The predominance of NTZ cases (77%) and the limited number of patients on S1P-RM, DMF, and those with JCV GCN may limit the generalizability of our findings to non-NTZ therapies. In addition, the fixed 12-month follow-up may fail to capture late functional recovery or delayed MS reactivation. Finally, outcome assessment through the mRS provides a relatively coarse evaluation and may not fully capture more subtle sequelae or their consequences on daily functioning.

Our findings reinforce the importance of early PML detection, premorbid functional status, and CSF JCV load as key prognostic indicators in MS-associated PML. While PML-IRIS appears to be associated with more favorable outcomes, neither PlEx nor corticosteroid use significantly influenced prognosis. These results underscore the complexity of managing iatrogenic PML and highlight the need for individualized therapeutic strategies and prospective studies to refine prognostic models and optimize care.

## Appendix Coinvestigators

**Table:**

Name	Location	Role	Contribution
Krämer Markus, MD	Department of Neurology, Alfried Krupp von Bohlen und Halbach Hospital, 45,117, Essen, Germany	Site investigator	Acquisition of data
Pompsch Mosche, MD	Department of Neurology, Alfried Krupp von Bohlen und Halbach Hospital, Alfried-Krupp-Str. 21, 45,117, Essen, Germany	Site investigator	Acquisition of data
Pallix-Guyot Maud, MD	Service de Neurologie et Unité Neurovasculaire, Centre Hospitalier Régional d'Orléans, 45,000 Orléans, France	Site investigator	Acquisition of data
Kume Kodai, MD, PhD	Department of Epidemiology Research Institute for Radiation Biology and Medicine, Hiroshima University, Japan	Site investigator	Acquisition of data
Misu Tatsuro, MD, PhD	Department of Neurology, Tohoku University Hospital, Japan	Site investigator	Acquisition of data
De Rossi Nicola, MD	Regional Multiple Sclerosis Center, ASST–Spedali Civili di Brescia, Montichiari, Italy	Site investigator	Acquisition of data
Gerevini Simonetta, MD	Department of Neuroradiology, Papa Giovanni XXIII Hospital, Bergamo, Italy	Site investigator	Acquisition of data
Ayzenberg Ilya, MD	Department of Neurology St. Josef-Hospital, Ruhr University Bochum, Germany	Site investigator	Acquisition of data
Schroeder Christoph, MD	Department of Neurology St. Josef-Hospital, Ruhr University Bochum, Germany	Site investigator	Acquisition of data
Marousset Aude, MD	Centre hospitalier régional universitaire de Tours, Hôpital Bretonneau, Service de neurologie, Tours, France	Site investigator	Acquisition of data
Martin-Blondel Guillaume, MD, PhD	Service des Maladies Infectieuses et Tropicales, Centre Hospitalier Universitaire de Toulouse, Toulouse, France	Site investigator	Acquisition of data
Jean-Christophe Ouallet, MD, PhD	Service de Neurologie, Pôle des Neurosciences Cliniques, CHU de Bordeaux Pellegrin Tripode, France	Site investigator	Acquisition of data
Vlad Benjamin, MD	Department of Neurology, University Hospital Zurich and University of Zurich, Zurich, Switzerland	Site investigator	Acquisition of data
Kana Veronika, MD, PhD	Department of Neurology, University Hospital Zurich and University of Zurich, Zurich, Switzerland	Site investigator	Acquisition of data
Heuer Christine, MD	Department of Neurology, University Hospital Zurich and University of Zurich, Zurich, Switzerland	Site investigator	Acquisition of data
Jelcic Ilijas, MD, PhD	Department of Neurology, University Hospital Zurich and University of Zurich, Zurich, Switzerland	Site investigator	Acquisition of data
Derfuss Tobias, MD	Neurologic Clinic and Policlinic and Research Center for Clinical Neuroimmunology and Neuroscience, Departments of Medicine, Biomedicine, and Clinical Research, University Hospital Basel, University of Basel, Basel, Switzerland	Site investigator	Acquisition of data
Karypidis Panajotis, MD	Neurologic Clinic and Policlinic and Research Center for Clinical Neuroimmunology and Neuroscience, Departments of Medicine, Biomedicine, and Clinical Research, University Hospital Basel, University of Basel, Basel, Switzerland	Site investigator	Acquisition of data
Romero-Pinel Lucia, MD	Neurology Department, Multiple Sclerosis Unit, Hospital Universitari de Bellvitge, IDIBELL, Barcelona, Spain	Site investigator	Acquisition of data
Bakirtzis Christos, MD, PhD	Multiple Sclerosis Center, Second Department of Neurology, Aristotle University of Thessaloniki, Greece	Site investigator	Acquisition of data
Nikolaos Grigoriadis, MD, PhD	Multiple Sclerosis Center, Second Department of Neurology, Aristotle University of Thessaloniki, Greece	Site investigator	Acquisition of data
McGraw Corey, MD	Neurology, State University of New York Upstate Medical University, Syracuse	Site investigator	Acquisition of data
Piñar Morales Raquel, MD	Servicio de Neurología, Hospital Clínico Universitario San Cecilio, Granada, Spain	Site investigator	Acquisition of data
Laplaud, David, MD, PhD	Nantes University Hospital, Neurology Department, CRC-SEP, Nantes University, INSERM, CIC 1413, Center for Research in Transplantation and Translational Immunology, UMR 1064, F-44000, Nantes, France	Site investigator	Acquisition of data

## Glossary

DMF: dimethyl fumarate
DMT: disease-modifying therapy
EDSS: Expanded Disability Status Scale
IQR: interquartile range
IRIS: immune reconstitution inflammatory syndrome
JCV: JC virus
mRS: modified Rankin Scale
MS: multiple sclerosis
NTZ: natalizumab
PML: progressive multifocal leukoencephalopathy
